# Phylogenetic and Metagenomic Analyses of Substrate-Dependent Bacterial Temporal Dynamics in Microbial Fuel Cells

**DOI:** 10.1371/journal.pone.0107460

**Published:** 2014-09-09

**Authors:** Husen Zhang, Xi Chen, Daniel Braithwaite, Zhen He

**Affiliations:** 1 Department of Civil and Environmental Engineering, Virginia Polytechnic Institute and State University, Blacksburg, Virginia, United States of America; 2 Department of Civil, Environmental, and Construction Engineering, University of Central Florida, Florida, United States of America; 3 Institute for Genomics and Systems Biology, Argonne National Laboratory, Chicago, Illinois, United States of America; Missouri University of Science and Technology, United States of America

## Abstract

Understanding the microbial community structure and genetic potential of anode biofilms is key to improve extracellular electron transfers in microbial fuel cells. We investigated effect of substrate and temporal dynamics of anodic biofilm communities using phylogenetic and metagenomic approaches in parallel with electrochemical characterizations. The startup non-steady state anodic bacterial structures were compared for a simple substrate, acetate, and for a complex substrate, landfill leachate, using a single-chamber air-cathode microbial fuel cell. Principal coordinate analysis showed that distinct community structures were formed with each substrate type. The bacterial diversity measured as Shannon index decreased with time in acetate cycles, and was restored with the introduction of leachate. The change of diversity was accompanied by an opposite trend in the relative abundance of *Geobacter*-affiliated phylotypes, which were acclimated to over 40% of total Bacteria at the end of acetate-fed conditions then declined in the leachate cycles. The transition from acetate to leachate caused a decrease in output power density from 243±13 mW/m^2^ to 140±11 mW/m^2^, accompanied by a decrease in Coulombic electron recovery from 18±3% to 9±3%. The leachate cycles selected protein-degrading phylotypes within phylum Synergistetes. Metagenomic shotgun sequencing showed that leachate-fed communities had higher cell motility genes including bacterial chemotaxis and flagellar assembly, and increased gene abundance related to metal resistance, antibiotic resistance, and quorum sensing. These differentially represented genes suggested an altered anodic biofilm community in response to additional substrates and stress from the complex landfill leachate.

## Introduction

Microbial fuel cells (MFCs) rely on anode-respiring bacteria that perform extracellular electron transfer [1]–[4]. Understanding the structure and genetic potential of anodic communities is one of the key factors to improve the MFC performance towards practical applications such as wastewater treatment, nutrient recovery, powering remote electronics, and producing value-added chemicals [5]–[7]. There has been strong interest in identifying steady-state microbial communities influenced by factors such as substrate, temperature, anode set potential, external resistance, and inoculum source [8]–[13].

While stable anode communities are important to MFC performance, changes of substrate and potential introduction of inhibitors occur when the composition and concentration of the anode feeding stream varies over time, which is commonly seen with actual waste streams. Elucidating temporal dynamics of the anode microbial communities at non-steady-state will be valuable because component fluctuations in waste streams will likely affect community structures. One example of such streams is landfill leachate with varying compositions depending on the age and type of solid wastes. Landfill leachate also contains high fraction of non-biodegradable organic matter, ammonia, and heavy metals [14], among which free ammonia and heavy metals are well known constituents that inhibit microbial activities [15]. Although MFCs have been applied to treat landfill leachate with simultaneous electricity generation [16]–[18], it is unclear how the anode communities respond to complex components in the leachate.

Microbial community analysis for MFCs has largely focused on targeting the 16S rRNA-gene and its product. Indeed, recent developments in barcoded high-throughput sequencing has enabled combining multiple samples in one sequencing run, facilitating sophisticated experimental designs [19]. However, using the 16S rRNA based phylogeny has well-known limitations in inferring genome contents and community functions [20]. Metagenomic shotgun sequencing complements the 16S rRNA-gene sequencing by directly measuring protein-coding genes and metabolic pathways [21]–[23]. Because no PCR is involved in generating libraries, metagenomics-based approaches do not have PCR bias. Metagenomic surveys would be especially valuable for landfill leachate containing highly complex substrates and toxic compounds.

In this study, we aimed to investigate bacterial community temporal dynamics and functions on the MFC anode using 16S rRNA-gene sequencing and metagenomic shotgun sequencing alongside with electrochemical characterizations. The MFC was first acclimated to acetate then to acetate-amended landfill leachate. Time-related bacterial community structures were reconstructed at each operating cycle for the two feeding streams. Differentially represented genes and pathways were identified and compared.

## Materials and Methods

### Experimental Setup

The MFC performance and microbial community change over time was monitored in a longitudinal design. A single-chamber, air-cathode MFC was constructed based upon an established design [5]. The diameter was 3.2 cm with a working volume of 40 mL. The anode and the cathode were both made of wet-proof carbon cloth (Fuel Cell Earth, Wakefield, MA). The cathode was coated with 0.5 mg/cm^2^ of Pt as a catalyst. Both electrodes had a projected surface area of 8 cm^2^. The MFC was inoculated with waste activated sludge from Eastern Water Reclamation Facility (Orlando, Florida, USA). The autoclaved feed medium was based on published recipes [24]. The MFC was acclimated for 30 days before reaching a stable peak voltage of 0.55 V with an external resistor of 5 kΩ. Then, an experimental fed-batch run of 12 cycles started with acetate as the feed from cycle 1 to 7, followed by acetate-amend leachate from cycle 8 to 12. The landfill leachate, whose components listed in Table S1, was obtained from a conventional landfill for municipal solid waste in Central Florida. The leachate was sterilized by autoclaving before use. The acetate-amended landfill leachate was made by mixing equal volumes of deionized water-diluted leachate (1/10 of the original strength) with the acetate-only medium. The MFC chamber was purged with nitrogen gas for 5 min at the beginning of each new cycle to maintain anoxic conditions.

The voltage (*E*) and current (*I*) were monitored at 30-min intervals using a digital multimeter with a data acquisition system (Keithley, Model 2700, Cleveland, OH, USA). Power (*P*) was calculated as *I*×*E*, and normalized to the anode surface area. The Coulombic efficiency (CE) was calculated as CE  =  (*C_p_*/*C_T_*)×100%, where C_p_ is the coulombs recovered as current and C_T_ is the total consumed coulombs [25]. The Coulombic recovery (CR) was calculated based on total input coulombs. The chemical oxygen demand (COD) concentration was measured according to standard methods [26]. We employed a “single cycle” method to generate polarization curves [27], in which voltage and current were measured within a short time period of 3 hours under varying external resistance from 51 kΩ to 0.1 kΩ.

### Bacterial community analysis of pyrosequences

Anode-attached biomass was harvested at each cycle using a sterile pipette tip, and stored immediately at −80°C until DNA extraction. DNA was extracted with a modified phenol-chloroform method [28]. Briefly, a frozen biomass (ca. 50 mg) was mixed with 500 µl of extraction buffer containing 200 mM Tris, 200 mM NaCl, 20 mM EDTA, 3% SDS, and 300 µl of 0.1-mm glass beads (BioSpec, OK, USA). Cells were mechanically disrupted and chemically lysed by bead beating for 30 sec. DNA was extracted with phenol:chloroform:isoamyl alcohol (500 µl, 25∶24∶1, Sigma Aldrich), followed by ethanol precipitation. DNA was evaluated by spectroscopic methods (NanoDrop 2000, Thermo Scientific) and agarose gel electrophoresis. Bacterial 16S rRNA genes were PCR-amplified with barcoded forward primer 27F and reverse primer 338R [29]. The PCR condition was 94°C for 1 min, and 30 cycles of 94°C for 30 s, 53°C for 45 s, and 72°C for 90 s, followed by a final extension at 72°C for 10 min. Amplicons from 0.5 µl, 1 µl, and 2 µl of DNA templates were combined, purified by agarose gel electrophoresis, and eluted with the Qiagen MinElute Kit.

Pyrosequencing was performed at Engencore (University of South Carolina) single directionally with ‘Lib-A’ adaptors. Sequences were filtered based on Phred quality scores (q), maximum consecutive low-quality base calls (r), and minimum fraction of consecutive high-quality bases in a sequence (p), as specified in the QIIME version 1.7 software [30]. Chimeric sequences (0.9% of total reads) were identified with usearch61 [31] and removed from downstream analysis. High-quality, non-chimeric sequences were binned into operational taxonomic units (OTUs) at 97% with uclust [31]. Taxonomy was assigned using a Naïve Bayesian Classifier at RDP [32]. Shannon diversity index was calculated with QIIME. Principle coordinate analysis (PCoA) of communities was based on the weighted UniFrac distances [33] calculated from branch lengths of a phylogenetic tree [34]. Significance of PCoA was tested by a permutational multivariate ANOVA method [35].

### Metagenomic shotgun sequencing

Four DNA samples, duplicate for each substrate, were extracted at cycle 6 and 7 (acetate), and cycles 11 and 12 (leachate). The extracted genomic DNA was mechanically sheared with the Bioruptor system. Libraries were prepared using the Ion Xpress fragment library prep kit, and quantified using a bio-analyzer. Sequencing was performed on the Ion Torrent PGM for the 200 bp chemistry with the Ion 316™ chip kit. Standard quality control was performed followed by additional sequence quality filtering using the analysis pipeline at MG-RAST [36]. The filtered metagenomic reads were searched against the protein database at Kyoto Encyclopedia of Genes and Genomes (KEGG) [37] with a maximum E-value of e^−20^ and minimum identify cutoff of 60%. The KEGG orthologs were compared among treatment groups (acetate versus leachate). Differentially represented gene families were identified by two-sided Welch’s *t*-test.

## Results

### Power generation, Coulombic efficiencies, and polarization curves

**Figure 1:**
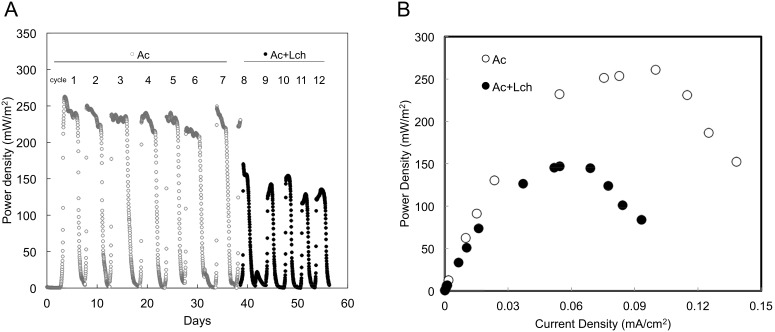
MFC performance. (**A**) Power generation in fed-batch mode with acetate (Ac, cycle 1 to 7) and a mixture of acetate/leachate (Ac+Lch, cycle 8 to 12) as the carbon source. (**B**) Polarization curves.

The output power density reached 243±13 mW/m^2^ for acetate-fed cycle 1 to cycle 7, and dropped for 42% to 140±11 mW/m^2^ soon after the introduction of leachate (Fig. 1A). The difference in maximum power density was evident based on the polarization curves (Fig. 1B). The Coulombic efficiency in acetate cycles (20±2%) was not significantly different from that in leachate cycles (15±5%) (*P* = 0.064, unpaired *t*-test, Table 1). The Coulombic recovery, calculated as the fraction of input electrons recovered as electric current, was significantly higher for acetate than leachate cycles (18±3% versus 9±3%, *P*<0.001).

**Table 1:** Fed-batch cycle start and end CODs, and Coulombic recoveries in the electric current as a percentage of consumed or input electrons.

Substrate	Cycle	COD (mg/L)		Coulomb		Coulombic Efficiency	Coulombic Recovery
		Start	End	As current	In removed COD	% of *consumed* electrons recovered as Coulomb	% of *input* electrons recovered as Coulomb
Acetate(Ac)	1	1998	140	189	896	21%	20%
	2	1993	160	183	884	21%	19%
	3	1994	139	206	895	23%	21%
	4	1992	200	172	865	20%	18%
	5	1990	300	145	815	18%	15%
	6	1990	162	179	882	20%	19%
	7	1988	180	135	872	15%	14%
						20% ± 2%	18% ± 3%
Acetate-amended leachate(Ac+Lch)	8	1484	267	75	587	13%	10%
	9	1304	553	23	362	6.2%	3.6%
	10	1304	524	67	376	18%	11%
	11	1302	592	63	342	18%	10%
	12	1300	650	61	314	20%	10%
						15% ± 5%	9% ± 3%

### Substrate-dependent bacterial temporal dynamics

**Figure 2:**
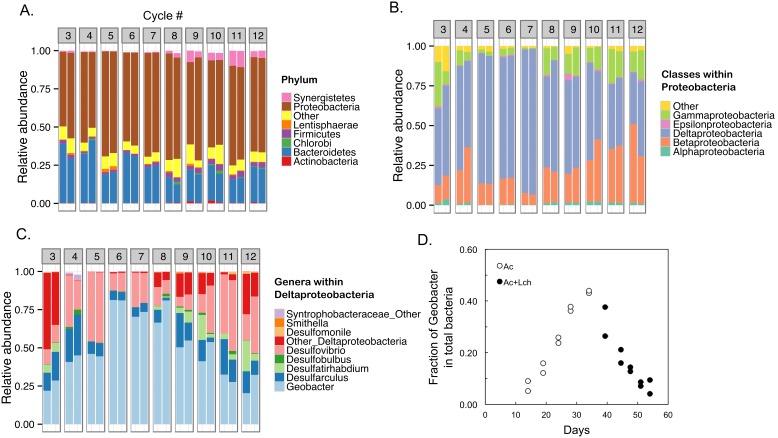
Bacterial temporal dynamics in the MFC. (**A**) Bacterial phyla composition as a function of MFC cycles that were labeled in Figure 1. Duplicate community compositions were shown for cycles 3 to 12. OTUs less than 0.1% of total bacteria were excluded from the plot. (**B**) Taxonomic breakdown for Proteobacteria, the most abundant phyla. (**C**). Further taxonomic breakdown of Deltaproteobacteria to the genus level. (**D**). The proportions of *Geobacter* at different time points.

At phylum level, Proteobacteria dominated anode communities, followed by Bacterioidetes (Fig. 2A). When leachate was introduced at cycle 8, members of Synergistetes (genera *Aminiphilus* and *Cloacibacillus*) started to appear. The most abundant class within Proteobacteria was Deltaproteobacteria (Fig. 2B). Betaproteobacteria started to increase when leachate was introduced. The relative abundance of *Geobacter* generally increased during the acetate-fed cycles then started to decrease in the leachate-fed cycles, measured either as a fraction of Deltaproteobacteria (Fig. 2C) or total Bacteria (Fig. 2D). At the OTU level, the most abundant *Geobacter*-affiliated OTU was classified as *Geobacter lovleyi* strain SZ, a freshwater acetate oxidizing, metal reducing bacterium [38].

**Figure 3:**
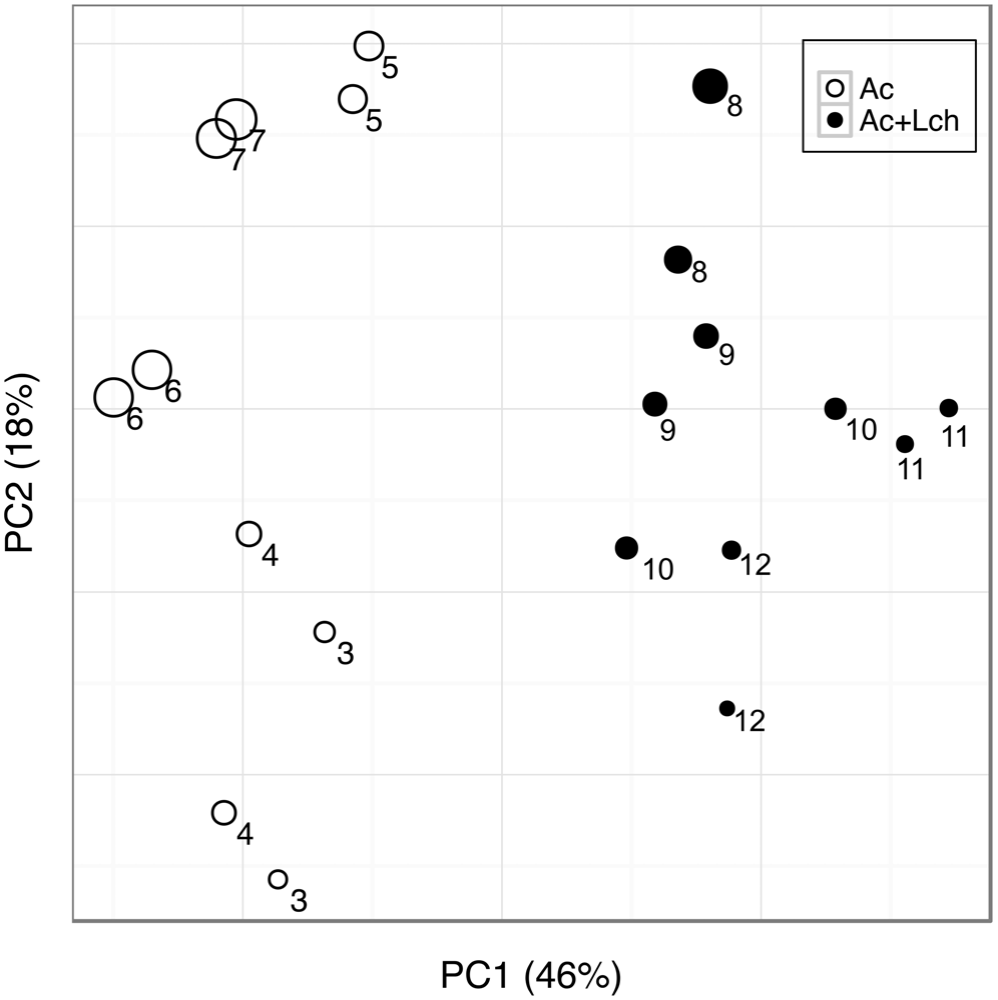
Principal coordinate analysis based on weighted UniFrac distances. The cycle number (3 through 12) is labeled next to data points. The size of circles was proportional to the relative abundance of *Geobacter*. Ac: acetate, Ac+Lch: acetate-amended leachate.

**Figure 4:**
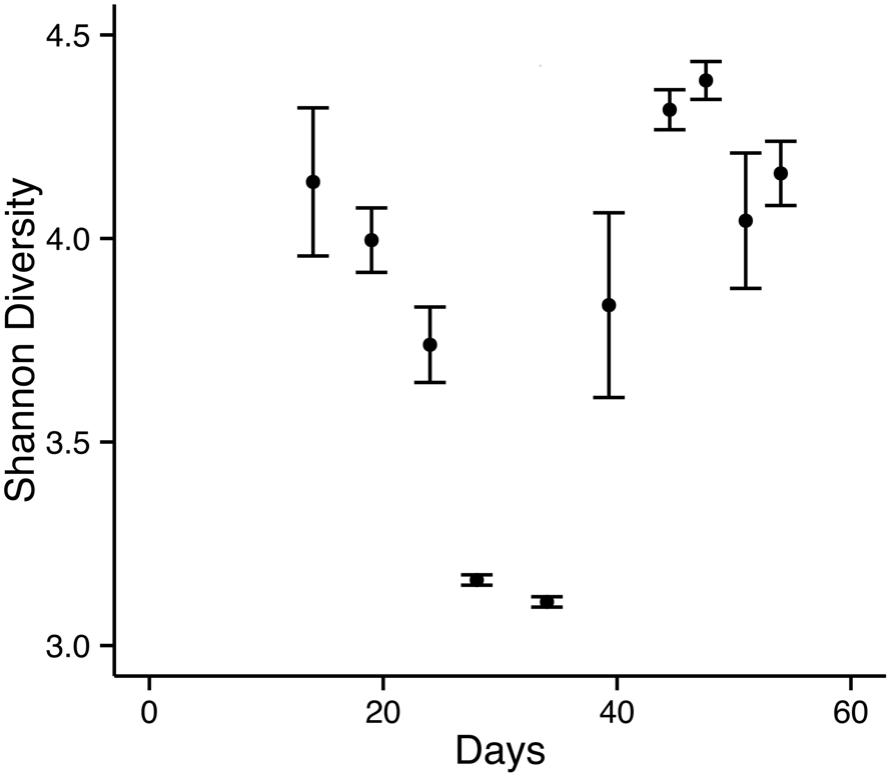
Change of community alpha diversity (measured as Shannon diversity) with time. Error bars represent standard error of the mean (n = 2).

To understand microbial community structure change over time, we measured Shannon diversity index for community richness and evenness, and used UniFrac distances as a measure of how similar or different the communities are among cycles. Principal coordinate analysis (PCoA) based on weighted UniFrac distances showed a clear separation between acetate-fed and leachate-fed communities along the first two axes that explained 46% and 18% of data variations, respectively (Fig. 3). Leachate as a significant source of community variation was confirmed by a permutational multivariate analysis of variance (PERMANOVA, *P*<0.001). Interestingly, the alpha diversity of microbial communities, measured by Shannon diversity index, decreased during the acetate-only cycles, and increased during the leachate cycles (Fig. 4). The increase of Shannon index in leachate cycles over acetate cycles was significant (*P* = 0.005). In general, a low Shannon index was associated with a high percentage of *Geobacter* (Fig. 2D and Fig. 4). This is consistent with low diversity due to one genus (*Geobacter*) becoming dominant in the community.

### Metagenomics of acetate-fed versus leachate-fed communities

**Figure 5:**
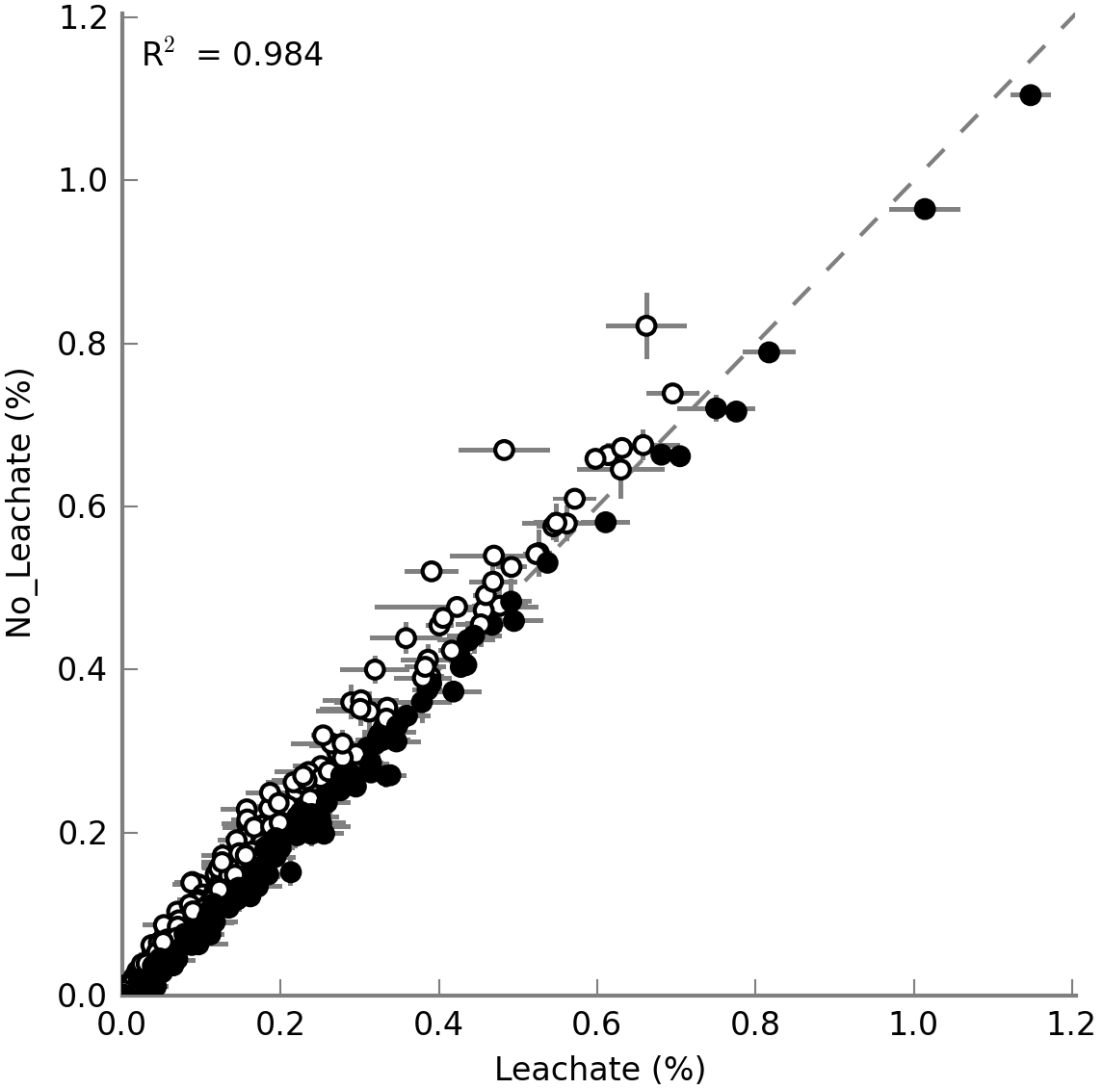
The proportions of KEGG Orthologs (KO) in the no-leachate and leachate-metagenomes plotted on the y and x axes, respectively. Data points above the dashed line were KOs that were more abundant in no-leachate communities. Data points below the dashed line were KOs that were more abundant in leachate-fed communities.

To investigate microbial community genetic potentials, and genes/pathways differentially affected by the feeding streams, we sequenced metagenomes of acetate (n = 2) and leachate-fed (n = 2) communities. A total of 1,533,922±563,725 reads per sample (n = 4) was obtained, representing 351±136 Mbp of data. The average read length was 228 bp. We analyzed the proportions of mapped KEGG Orthologs (KO) in the no-leachate and leachate-metagenomes (Fig. 5). Despite the overall correlation (R^2^ = 0.93) of metagenomes under acetate- and leachate-fed conditions, distinct genes and pathways were differentially represented in communities with each feed. Data points above the dashed line represented genes that were more abundant in the no-leachate communities (Fig. 5).

**Figure 6:**
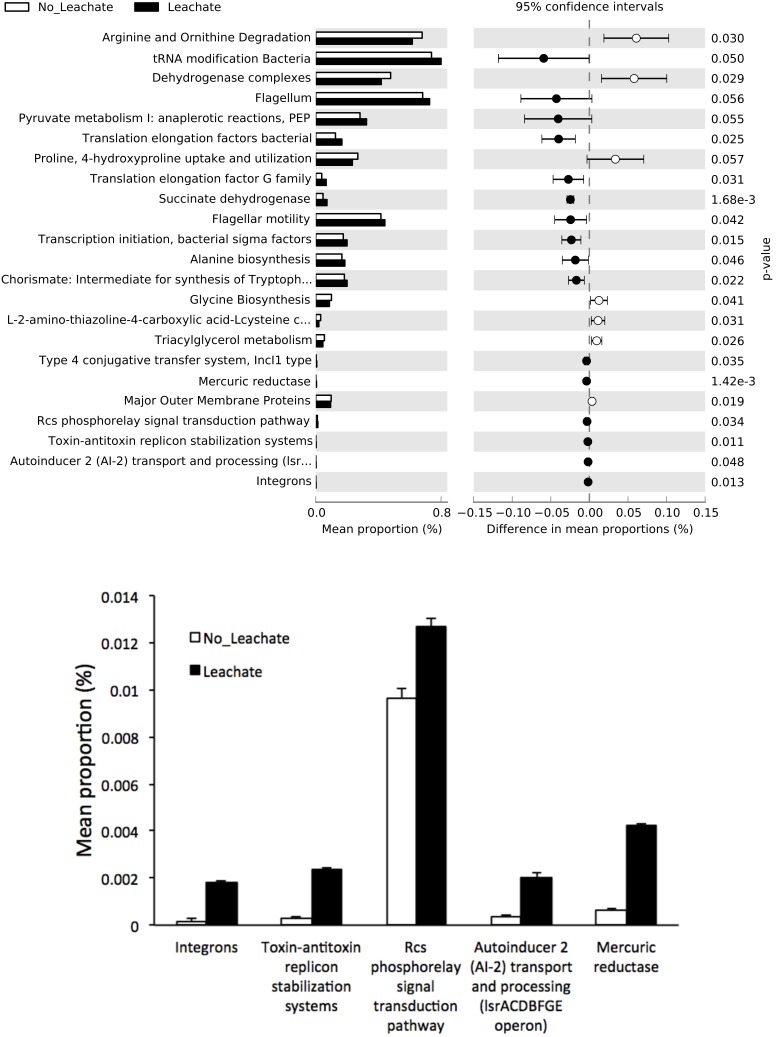
Differentially represented KOs in no-leachate and leachate metagenomes. The difference was shown in confidence intervals and *P* values based on the Welch’s *t*-test. (**A**): All differentially regulated KOs, and (**B**): Those differentially regulated KOs with a small effect size.

Metagenomic functional categories representing genes important for metabolisms including arginine and ornithine degradation, dehydrogenase complexes, succinate dehydrogenase, and several amino-acid biosynthesis were differentially represented with respect to the substrate (Fig. 6A). The plot of confidence intervals provided direction and size of treatment effects, and *P* values showed statistical significance of the treatments (Fig. 6A). A significant increase of cell motility genes including bacterial chemotaxis, flagellum, and flagellar assembly in the leachate-fed communities suggested that bacteria might be actively accessing substrates and/or adjusting their locations. Leachate also increased genes for heavy metal resistance, such as *merA*, the mercuric reductase gene, the antibiotic resistance related integrons [39], quorum sensing related autoinducer 2 operon [40], and Rcs phosphorelay signal transduction pathway (Fig. 6B). These resistance and communication related genes have been implicated in biofilm development [41].

## Discussion

The acetate-amended leachate-fed MFC produced higher power densities than several recent studies on leachate-fed MFCs [17], [18]. The Coulombic efficiency from the acetate cycles was similar to other fixed-resistance bioelectrochemical systems [42]. Compared with the acetate cycles, the leachate cycles produced lower power (and current) with lower COD removal, leading to a similar Coulombic efficiency (Table 1). The low COD removal in leachate was likely due to a large fraction of non-biodegradable organic matter in the landfill leachate [14].

The most abundant *Geobacter*-affiliated OTU was classified as *Geobacter lovleyi* strain SZ, which apparently came from the waste activated sludge inoculum. Its abundance dynamically changed with both the feed medium as well as the operating cycles. *G. lovleyi* has been shown to reduce tetrachloroethylene (PCE) with an graphite electrode as the electron donor, but its ability to serve as an anode-respiring bacterium was once considered to be limited [43]. We found that *G. lovleyi* could be acclimated to as high as over 40% in acetate-fed MFC. Whether this organism uses acetate as the electron donor would need to be investigated by labeling techniques such as stable isotope probing as demonstrated in other systems [44], [45]. Its decline in leachate-fed cycles might be due to toxicity from leachate components such as heavy metals. The second most abundant *Geobacter*-affiliated OTU was *Geobacter* sp. strain CLFeRB, a freshwater acetate-oxidizing, iron-reducing bacterium that could methylate mercury [46]. Future experiments will need to be conducted to assess how different reactor configurations affect community changes after leachate additions.

The alpha diversity (Shannon index) of anodic bacterial communities decreased during acclamation with acetate, then increased when leachate was fed into the MFC. It is conceivable that additional substrates in the leachate, such as proteins, allowed a more diverse community to colonize the anode. For example, an increase of members of *Aminiphilus* and *Cloacibacillus* (phylum Synergistetes) suggested that bacterial communities responded to substrate availability in the leachate. Both genera harbor amino-acid degrading anaerobes [47], [48]. Substrate type was found to be the driver for microbial community structures in anaerobic digestion systems [49]. It is unclear why Bacteroidetes fluctuated but persisted in the MFC as the second most abundant phylum (Fig. 2A), although members of Bacteroidetes were also found to be abundant on anodes with different sources of inoculum [11]. High microbial diversity has been well recognized as key to ensure robust ecosystem functions [50]. In particular, it was reported that anaerobic digesters with higher community diversity functioned more efficiently [51]. In our system, the lower power production observed from acetate to leachate transition could be due to the growth inhibition of the anode-respiring bacterium *G. lovleyi* from leachate components. Additional acclamation may be needed for more efficient power generation from the landfill leachate.

## Supporting Information

Table S1 **Chemical parameters of the leachate used in this study.** Data represent averages from three sampling dates in 2010.
(PDF)
