# Supplementary material for: Phylogenetic and Metagenomic Analyses of Substrate-Dependent Bacterial Temporal Dynamics in Microbial Fuel Cells
Source: PLoS One. 2014 Sep 9;9(9):e107460. doi: 10.1371/journal.pone.0107460 (PMC4159341; doi:10.1371/journal.pone.0107460)
Supplement: Table S1 — Chemical parameters of the leachate used in this study. Data represent averages from three sampling dates in 2010. (PDF) [file pone.0107460.s001.pdf]

Table S1. Chemical parameters of the leachate used in this study. Data represent averages from three sampling dates in 2010.

Components	Concentration (mg/L except for pH)
pH	7.7
Total Kjeldahl Nitrogen	1690
Total Phosphorus	9.6
Total Phenolics	7.3
Total Dissolved Solids	12700
Total Suspended Solids	216
Chloride (Cl)	2650
Chemical Oxygen Demand	10200
Biochemical Oxygen Demand	3560
Chromium (Cr)	0.3
Cobalt (Co)	0.1
Copper (Cu)	0.02
Manganese (Mn)	0.8
Zinc (Zn)	0.4
Cadmium (Cd)	0.1
Lead (Pb)	1.1
Selenium (Se)	14.8
Silver (Ag)	0.2
Mercury (Hg)	0.1
